# A potential mechanism of miana (*Coleus scutellariodes*) and quercetin via NF-κB in *Salmonella typhi* infection

**DOI:** 10.1016/j.heliyon.2023.e22327

**Published:** 2023-11-14

**Authors:** Ade Rifka Junita, Firdaus Hamid, Budu Budu, Rosdiana Natzir, Yusmina Hala, Gemini Alam, Rosana Agus, Burhanuddin Bahar, Ahmad Syukri, Muhammad Reza Primaguna, Ressy Dwiyanti, Andini Febrianti, Muhammad Sabir, Azhar Azhar, Mochammad Hatta

**Affiliations:** a Postgraduate School, Faculty of Medicine, Hasanuddin University, Makassar, Indonesia; b Molecular Biology and Immunology Laboratory, Faculty of Medicine, Hasanuddin University, Makassar, Indonesia; c Department of Microbiology, Faculty of Medicine, Hasanuddin University, Makassar, Indonesia; d Department of Ophthalmology, Faculty of Medicine, Hasanuddin University, Makassar, Indonesia; e Department of Biochemistry, Faculty of Medicine, Hasanuddin University, Makassar, Indonesia; f Department of Biology, Faculty of Sciences, State University of Makassar, Makassar, Indonesia; g Department of Pharmacognosy and Phytochemistry, Faculty of Pharmacy, Hasanuddin University, Makassar, Indonesia; h Department of Biostatistic, Faculty of Medicine, Hasanuddin University, Makassar, Indonesia; i Department of Internal Medicine, Faculty of Medicine, Hasanuddin University, Makassar, Indonesia; j Department of Medical Microbiology, Faculty of Medicine, Tadulako University, Palu, Indonesia; k Department of Forensic and Medicolegal, Faculty of Medicine, Hasanuddin University, Makassar, Indonesia; l Department of Pulmonology and Respiratory Medicine, Faculty of Medicine, Hasanuddin University, Makassar, Indonesia

**Keywords:** Anti-inflammation, Antibacterial, Miana (*Coleus scutellariodes*), Quercetin, Nuclear factor-kappa B (NF-κB), *Salmonella enterica* serovar typhi (S. typhi)

## Abstract

**Purpose:**

To prove the effect of Miana (M), Quercetin (Q), and the combination as an anti-inflammatory agent and Cefixime (C) as an antibiotic in Balb/c mice infected with *Salmonella enterica* serovar Typhi (S. Typhi) and related to the dynamics of NF-κB mRNA expression and NF-κB protein levels.

**Methods:**

A cohort study on male Balb/c mice with subjects consisted of 8 groups with 5 each group by administration of M, Q, M + Q, M + C, Q + C, M + Q + C, C only and sterile distilled water group as negative control. The statistical significance of the difference group was defined as *P* values less than 0.05.

**Results:**

Decreased mRNA expression of NF-κB, NF-κB protein levels, and bacterial load after administration of M + C, Q + C, or M + Q + C showed significant differences when compared to the negative control. The decline in NF-κB was stronger when M + Q + C was given compared to M, Q, M + Q, or C only.

**Conclusion:**

The effects of NF-κB suppression appear to be the same between the administration of M, Q and the M + Q when C added. However, the suppression of NF-κB was not significant without adding C.

## 1 Introduction

*Salmonella enterica* serovar Typhi (S. Typhi) is a cause of typhoid fever which can cause systemic inflammation and the incidence rate is still high in Indonesia and this is closely related to poor environmental sanitation and personal hygiene [1,2]. Besides that, the resistance rate of S. Typhi to antibiotics is still high and allows carriers to occur which are a source of transmission of S. Typhi through the digestive tract [3]. Previous studies has shown the transmission of S. Typhi that is resistant to multiple antibiotics (MDR) in Indonesia and Asia [2,4,5].

Miana (*Coleus scutellariodes*) is a tropical plant with a wide variety of plant colors or leaf colors. Miana has been widely used as traditional medicine in Indonesia [6]. Flavonoids can be assigned to numerous classes based on their chemical structures, including aurones, chalcones, anthocyanins, dihydrochalcones, isoflavonol, flavonol, dihydroflavonols, flavans, and proanthocyanidins, flavones and flavanones. Essential oils, saponins, carvacrol, tannins, flavonoids, thymol, and eugenol constitute some of the beneficial chemical compounds found in Miana, and the presence of its active components such alkaloids, flavonoids, and phenolic derivatives (polyphenols) can have antibacterial properties. [7, 8].

Through the innate immune response, Nuclear Factor Kappa Beta (NF-κB) is essential for the host's defense against microbial pathogen invasion. Numerous signaling pathways coming from various cellular receptors and sensors cause NF-κB to become active. Most pathogenic bacteria are particularly adept at controlling NF-κB activation, which they then use to trigger signals for numerous proinflammatory cytokines [9]. Quercetin is a derivative of flavonoids, where previous studies have shown the effect of Quercetin in inhibiting inflammatory processes [10,11].

In previous studies it was proved that giving Miana which contains flavonoids and Quercetin which is a derivative of flavonoids has an anti-inflammatory effects because it can inhibit the release of histamine, inflammatory cytokines. Inhibits leukocyte metabolic activity, and inflammatory cytokines release [[12], [13], [14], [15]] The anti-inflammatory effect of Quercetin and Miana can be mediated by inhibition of NF-κB activation and inflammatory cytokine release [16]. Miana also significantly reduced the levels of Vascular Endothelial Growth Factor (VEGF), Hypoxia Inducible Factor-1 alpha (HIF-1α), and reduced inflammation in *M. tuberculosis* infection [17,18].

The mechanism of action of Miana and Quercetin as anti-inflammatory in S. Typhi infection through suppression of NF-κB activity is an important part in reducing the inflammatory process.

This study was conducted to prove the effect of giving Miana, Quercetin and the combination as an anti-inflammatory in Balb/c mice infected with S. Typhi and related to the dynamics of NF-κB mRNA expression, NF-κB protein level, and bacterial load.

## 2 Materials and methods

This study is a prospective laboratory experimental study on Balb/c mice using a simple randomized design. The research was conducted at the Laboratory of Molecular Microbiology and Immunology, Faculty of Medicine, University of Hasanuddin Makassar. The research was carried out after obtaining approval for ethical clearance from the health research ethics committee of the Faculty of Medicine UNHAS Makassar, Indonesia, No 306/UN4.6.4.5.3U PP36/2023, dated May 12, 2023 according to Helsinki Declaration.

### 2.1 Experimental animals

The research samples were Balb/c mice, male, adults, healthy, 10–12 weeks of age, and 35–40 g of body weight. This study had 8 treatment groups and 5 mice per treatment. Miana was extracted according to a previous study [[17], [18], [19]]. The Miana administration at a dose of 510 mg/kg body weight for 14 days and/or the Quercetin (Nature Bell, USA) administration at a dose of 1000 mg/kg BW for 11 days after 24 h of inoculation of 2 × 10^4^ CFU/mL S. Typhi (0.2 mL) [[18], [19], [20]]. S. Typhi was provided by stock reference according to previous study [21,22]. Cefixime administration as a positive control at a dose of 4 mg/kg BW of Cefixime per day by the gastric tube for 5 days and the negative control was Balb/c mice given sterile distilled water through the gastric tube after 24 h of inoculating 2 × 10^4^ CFU/mL S. Typhi (0.2 mL) intraperitoneally [23].

Before being inoculated with S. Typhi, after 24 h post-inoculation and after complete Miana and/or Quercetin and/or Cefixime intervention were determined the NF-κB mRNA expression and NF-κB protein level of each mice group.

### 2.2 Examination of mRNA NF-κB mRNA expression

The RNA was extracted from the blood of the tail vein of mice. A 100 μl of fresh blood was added to 900 μl of “L6” solution consisting 120 g of Guanidium thiocyanate (GuSCN) (Sigma, cat no. G9277) in 100 ml of 0.1 M Tris HCl, pH 6.4, 22 ml 0.2 M Ethylene Diamine Tetra Acetate (EDTA) pH 8.0 and 2.6 g Triton X-100 (Merck, cat no 1.08603) with a final concentration of 50 mM Tris HCl, 5 M GuSCN, 20 mM EDTA, and 0.1 % Triton X-100. Subsequently, the mixture was centrifuged at 12,000 rpm and the sediment was added to a 20 μl diatom suspension consisting of 50 ml H_2_O and 500 μl of 32 % (w/v) “Celite” (Sigma, cat no. 22140). Moreover, 20 μl of this diatom suspension could bind 10 μg of tissue RNA, it was vortexed and centrifuged in a 1.5 ml Eppendorf tube at 12,000 rpm for 15 min. The supernatant was removed, and the sediment was washed by adding 1 ml of “L2” solution which consist of 120 g of GuSCN in 100 ml 0.1 M Tris HCl, pH 6.4. It was vortexed and centrifuged at 12,000 rpm for 15 min, the washing was repeated 2 times using an “L2” solution, and subsequently with 1 ml of 70 % ethanol 2 times and 1 ml of acetone. The resulting mixture was heated in a water bath at 56 °C for 10 min and 60 μl of “TE” solution consisting of 1 mM EDTA was added to 10 mM Tris HCL pH 8.0. Furthermore, it was vortexed and centrifuged at 12,000 rpm for 30 s, and incubated in the oven for 10 min at 56 °C. It was vortexed and re-centrifuged for 30 s at 12,000 rpm, and the supernatant was obtained. The supernatant from this process produced nucleotide extraction results and was stored at −80 °C before performing PCR analysis [20,24]. NF-κB mRNA expression was analyzed and determined by quantitative real-time PCR [[25], [26], [27]]. Detecting the NF-kβ mRNA expression using specific primers forward: CAGCTCTTCTCAAAGGAGCA and reverse: TCCAGGTCATAGAGAGGCTCA. GAPDH as Housekeeping gene using forward primer: GGTGCATGGCCGTTCTTA, and GAPDH reverse primer: TCGTTCGTTATCGGAATTAACC. PCR conditions are the initial stage of activation with a temperature of 95 °C for 45 s, followed by 30 cycles at a temperature of 95 °C for 30 s and 54 °C for 30 s for annealing [27]. qRT-PCR using SYBR Green qRT-PCR master mix kit, single stage. This protocol is optimized for real time PCR 96 well 0.1 ml of CFX Connect system instrument, Biorad Laboratories, USA. The NF-κB mRNA expression was assessed in fold change (fc) and using the 2^−ΔΔCT^ method.

### 2.3 Examination of NF-κB protein level

Serum obtained from the tail vein of Balb/c mice samples was used for determining the protein level of NF-κB. Each sample was duplicated to ensure the validity of the ELISA results. NF-κB was examined using sandwich ELISA mice NF-κB from KIT and read using ELISA Reader 270 (Biomerieux, France). Briefly, bring all reagents and samples to room temperature without additional heating and mix thoroughly by gently swirling before pipetting (avoid foaming). Bring the TMB Substrate up to 37 °C for 30 min prior to use. Add 100 μl of Standard, Blank, or Sample per well, cover with a plate sealer, and incubate for 90 min at 37 °C and aspirate to remove liquid then invert the plate and tap against clean absorbent paper. Wash each well 2 times and add 100 μl of 1x Biotinylated Detection Antibody working solution to each well, cover with a plate sealer, and gently agitate to ensure thorough mixing. Incubate for 60 min at 37 °C. Aspirate the liquid from each well and wash 3 times and wash by adding approximately 350 μl of 1x Wash Buffer using a squirt bottle, multi-channel pipette, manifold dispenser or automated washer. Allow each wash to sit for 1–2 min before completely aspirating.

After the last wash, aspirate to remove any remaining Wash Buffer then invert the plate and tap against clean absorbent paper. Horseradish Peroxidase (HRP)-Streptavidin in 100 μl was added. Aspirate the liquid from each well, then wash five times. Mix the working solution into each well, cover with a fresh plate sealer, and incubate for 30 min at 37 °C. 90 μl of TMB Substrate solution should be added to each well. Then, a fresh plate sealer should be applied. Incubate for 10–20 min at 37 °C. Until optimal color development has been achieved, shield from light and periodically check. To each well, add 50 μl of Stop Solution. The blue color will instantly turn yellow. Gently tapping the plate will ensure thorough mixing if the color change does not appear to be uniform. Wells should receive the Stop Solution in the same pattern and at the same time as the TMB Substrate solution. Analyze the optical density (OD) of each well immediately using a microplate reader set to 450 nm. NF-κB protein levels are expressed in picograms (pg/ml) [27].

### 2.4 Bacterial load

The technique previously described was used to measure the bacterial load. A sterile syringe was used to remove the peritoneal fluid, which was then centrifuged for 3 min at 3000 rpm. Following a gradient dilution (10^−2^ to 10^−7^), the supernatant was streaked onto a Salmonella Shigella agar (SS agar) plate in 100 μl to examine for S. Typhi. CFU counts on each plate were used to determine the number of bacteria present. The following formula was used to calculate the quantity of bacteria present: Bacterial load (CFU/ml) = 10^3^ x CFUs on plate/dilution [21,22].

### 2.5 Data management and analysis

The data obtained is processed and the results are displayed in the form of a narrative, table or graph. Statistical analysis using the SPSS 26 device with the normality test method for NF-κB gene mRNA expression and NF-κB protein levels, experimental animals were tested with the Shapiro Wilk test. Changes in the dynamics of NF-κB gene mRNA expression with NF-κB protein levels, between groups of experimental animals were tested by unpaired *t*-test. The dynamics of changes in NF-κB gene mRNA expression with bacterial load in each treatment group of experimental animals was tested by paired *t*-test. Different statistically significant, if *p* < 0.05.

## 3 Results

### 3.1 NF-κB mRNA expression after administration of miana, quercetin, and/or cefixime

NF-κB mRNA expression after administration of M, Q, and/or C in mice induced by S. Typhi shows that NF-κB mRNA expression was low before infection (6.24 ± 0.64 fc) and increased two folds significantly after infection (12.15 ± 0.61 fc) (*p* < 0.05). In the negative control group, NF-κB mRNA expression continued to increase significantly until the end of the observation (14.63 ± 0.39 fc) (*p* < 0.05), while NF-κB mRNA expression in the group that was given the M intervention (10.12 ± 0.69 fc) or Q (10.23 ± 0.52 fc) or the combination of M + Q (10.15 ± 0.43 fc) showed a significant decrease compared to the negative control (*p* < 0.05). There was no significant difference in decreasing NF-κB mRNA expression between the M or Q groups and the M + Q (*p* > 0.05).

Furthermore, the combination of M or Q with C as an antibiotic showed a significant decrease in NF-κB mRNA expression (7.96 ± 0.27 fc and 7.88 ± 0.32 fc) compared to the M or Q groups alone (*p* < 0.05). Likewise the M + Q + C group (7.97 ± 0.39 fc) showed a significant decrease compared to the M or Q or the M + Q administration (*p* < 0.05). In the administration of C as a positive control showed a decrease in NF-κB mRNA expression (8.50 ± 0.27 fc) but not as low as when given a combination of C with M or Q or M + Q (Table 1).

**Table 1:** NF-κB mRNA expression after administration of Miana, Quercetin, and/or Cefixime in mice induced by S. Typhi.

Group	NF-κB mRNA expression ± SD (fc)	*P* value
Before infection	6.24 ± 0.64	
Infection	12.15 ± 0.61	0.00^a^ (*p* < 0,05)
Group:	14.63 ± 0.39	0.00^b^ (*p* < 0.05)
Negative control		
Miana (M)	10.12 ± 0.69	0.00^c^ (*p* < 0,05)
Quercetin (Q)	10.23 ± 0.52	0.78^d^ (*p* > 0,05)
M + Q	10.15 ± 0.43	0.93^e^ (*p* > 0,05)
M + Cefixime (C)	7.96 ± 0.27	0.00^f^ (*p* < 0,05)
Q + C	7.88 ± 0.32	0.00^g^ (*p* < 0,05)
M + Q + C	7.97 ± 0.39	0.71^h^ (*p* > 0,05)
C	8.50 ± 0.27	0.03^i^ (*p* < 0,05)

a Comparison of Before infection vs Infection.

b Comparison of Negative control vs Infection.

c Comparison of M vs Negative control.

d Comparison of Q vs M.

e Comparison of Q + M vs M or Q.

f Comparison of M + C vs M alone.

g Comparison of Q + C vs Q alone.

h Comparison of M + Q + C vs Q + C.

i Comparison of C vs M + Q + C.

**Fig. 1:**
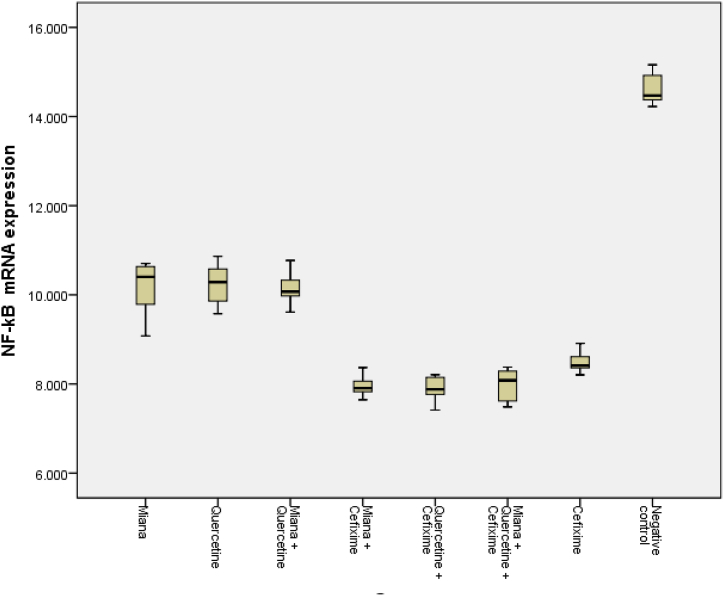
The comparison of NF-κB mRNA expression between various intervention of Miana, Quercetin, and/or Cefixime.

Fig. 1, the comparison of NF-κB mRNA expression between various interventions of M, Q, and/or C. Administration of M or Q or a combination of M + Q suppressed the similar NF-κB mRNA expression. Likewise, after administration of the combination M + C or Q + C or M + Q + C found almost the same to suppress the NF-κB mRNA expression. Meanwhile, the suppression of NF-κB mRNA expression after administration of C only was lower than the combination of M + C or Q + C or M + Q + C.

### 3.2 NF-κB protein level after administration of miana, quercetin, and/or cefixime

NF-κB protein level after administration of M, Q, and/or C in mice induced by S. Typhi shows that NF-κB protein level was low before infection (307.41 ± 37.35 pg/ml) and increased 2 times significantly after infection (585.88 ± 29.04 pg/ml) (*p* < 0.05).

In the negative control group, NF-κB protein level continued to increase significantly until the end of the observation (735.98 ± 22.92 pg/ml) (*p* < 0.05), while NF-κB protein level in the group that was given the M intervention (491.60 ± 40.58 pg/ml) or Q (497.02 ± 26.84 pg/ml) or the combination of M and Q (472.65 ± 31.55 pg/ml) showed a significant decrease compared to the negative control (*p* < 0.05). There was no significant difference in decreasing NF-κB protein level between the M or Q groups and the M + Q (*p* > 0.05).

Furthermore, the combination of M or Q with C as an antibiotic showed a significant decrease in NF-κB protein level (381.21 ± 18.50 pg/ml and 379.00 ± 12.88 pg/ml) compared to the M or Q groups alone (*p* < 0.05). Likewise the M + Q + C group (389.02 ± 14.76 pg/ml) showed a significant decrease compared to the M or Q or the M + Q administration (*p* < 0.05). In the administration of C as a positive control showed a decrease in NF-κB protein level (411.59 ± 11.61 pg/ml) but not as low as when given a combination of C with M or Q or M + Q (Table 2).

**Table 2:** NF-κB protein level after administration of Miana, Quercetin, and/or Cefixime in mice induced by S. Typhi.

Group	NF-κB protein level ± SD (pg/ml)	*P* value
Before infection	307.41 ± 37.35	
Infection	585.88 ± 29.04	0.00^a^ (*p* < 0,05)
Group:	735.98 ± 22.92	0.00^b^ (*p* < 0.05)
Negative control		
Miana (M)	491.60 ± 40.58	0.00^c^ (*p* < 0,05)
Quercetin (Q)	497.02 ± 26.84	0.81^d^ (*p* > 0,05)
M + Q	472.65 ± 31.55	0.43^e^ (*p* > 0,05)
M + Cefixime (C)	381.21 ± 18.50	0.01^f^ (*p* < 0,05)
Q + C	379.00 ± 12.88	0.00^g^ (*p* < 0,05)
M + Q + C	389.02 ± 14.76	0.29^h^ (*p* > 0,05)
C	411.59 ± 11.61	0.02^i^ (*p* < 0,05)

a Comparison of Before infection vs Infection.

b Comparison of Negative control vs Infection.

c Comparison of M vs Negative control.

d Comparison of Q vs M.

e Comparison of Q + M vs M or Q.

f Comparison of M + C vs M alone.

g Comparison of Q + C vs Q alone.

h Comparison of M + Q + C vs Q + C.

i Comparison of C vs M + Q + C.

**Fig. 2:**
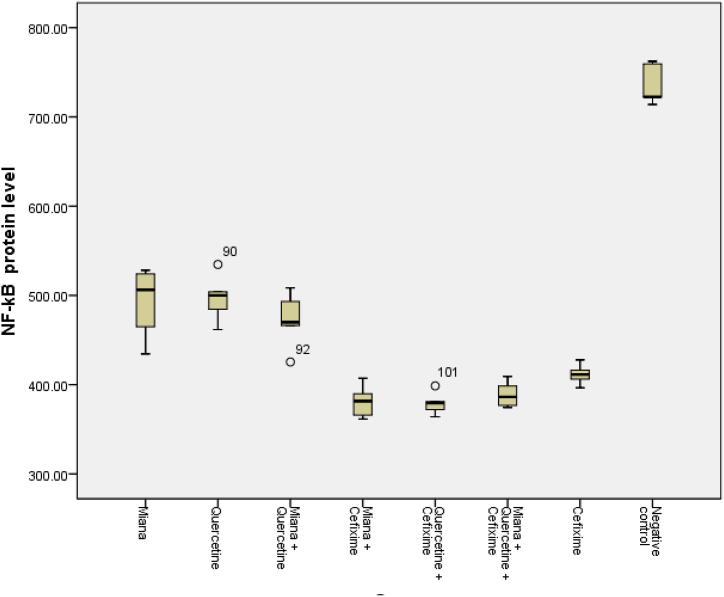
The comparison of NF-κB mRNA protein level various intervention of Miana, Quercetin, and/or Cefixime.

Similar to the ability of various interventions of M, Q, and/or C to suppress NF-κB mRNA expression after the intervention, the NF-κB protein level is shown in Fig. 2. The comparison of NF-κB protein levels between various interventions of M, Q, and/or C revealed that administration of M or Q or a combination of M + Q suppressed the similar NF-κB protein level. Likewise, after administration of the combination M + C or Q + C or M + Q + C found almost the same suppression of NF-κB protein level. Meanwhile, the suppression of NF-κB protein level after administration of C only was lower than the combination of M + C or Q + C or M + Q + C.

### 3.3 Bacterial load quantification

**Fig. 3:**
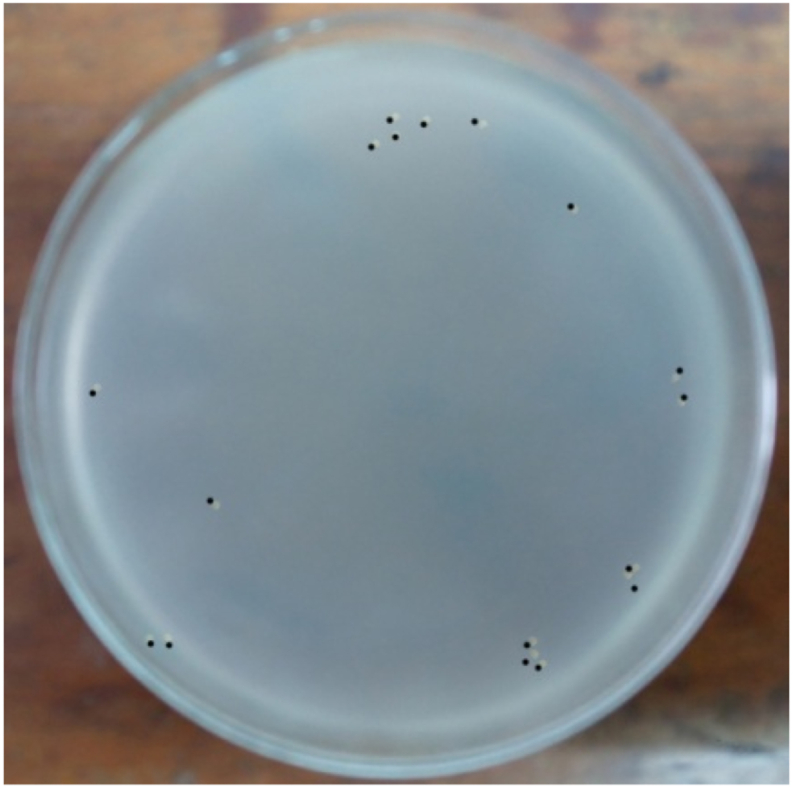
Bacterial growth inhibition by Miana or Quercetin and/or Cefixime intervention. Colony growth of S. Typhi after M.

**Fig. 4:**
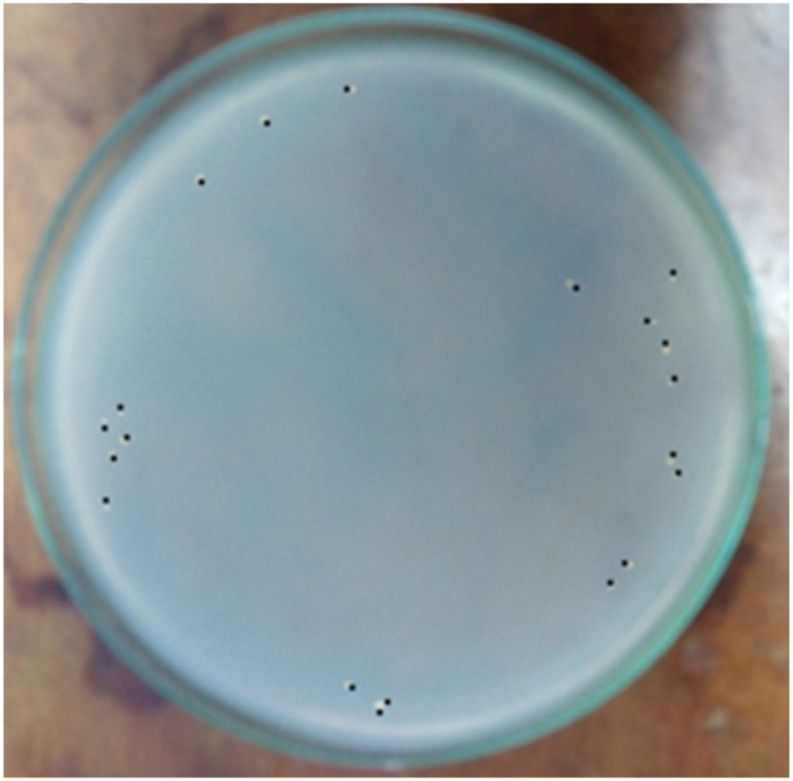
Bacterial growth inhibition by Miana or Quercetin and/or Cefixime intervention. Colony growth of S. Typhi after Q.

**Fig. 5:**
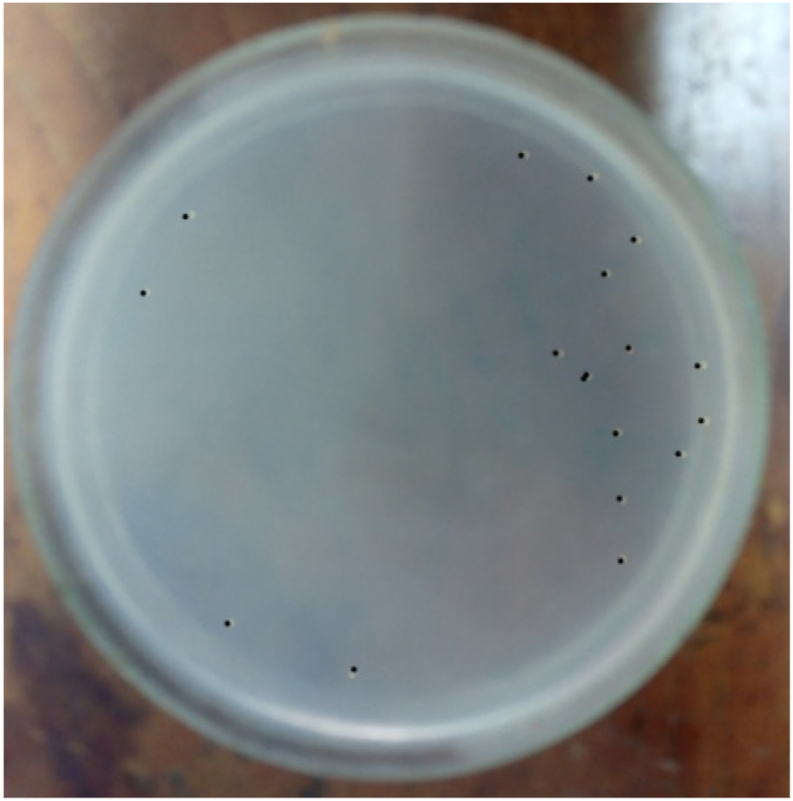
Bacterial growth inhibition by Miana or Quercetin and/or Cefixime intervention. Colony growth of S. Tyuphi after M + Q intervention.

**Fig. 6:**
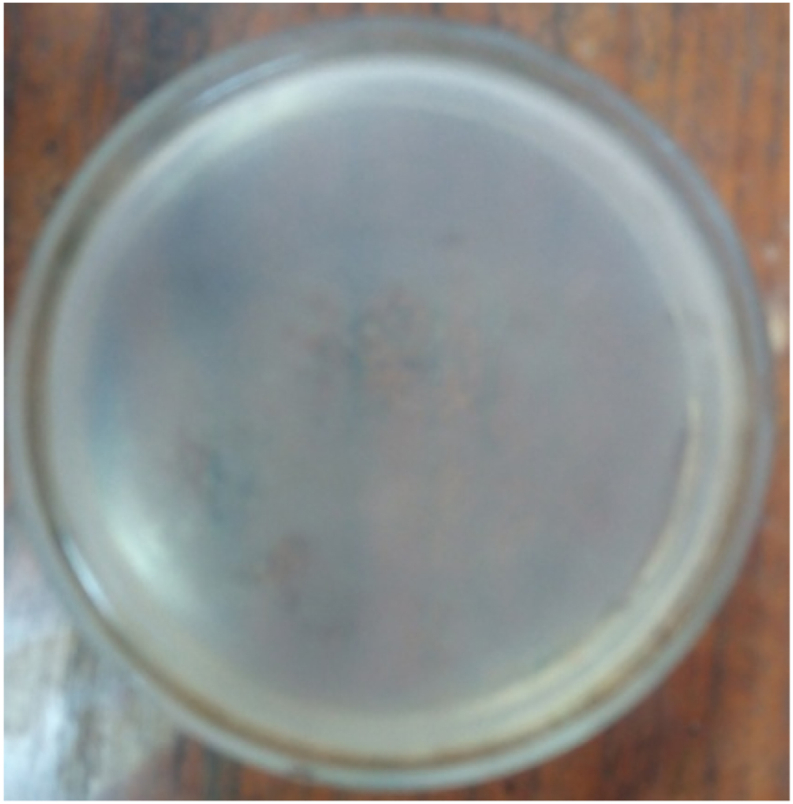
Bacterial growth inhibition by Miana or Quercetin and/or Cefixime intervention. No growth of S. Typhi after C or M + C or Q + C or M + Q + C intervention.

**Fig. 7:**
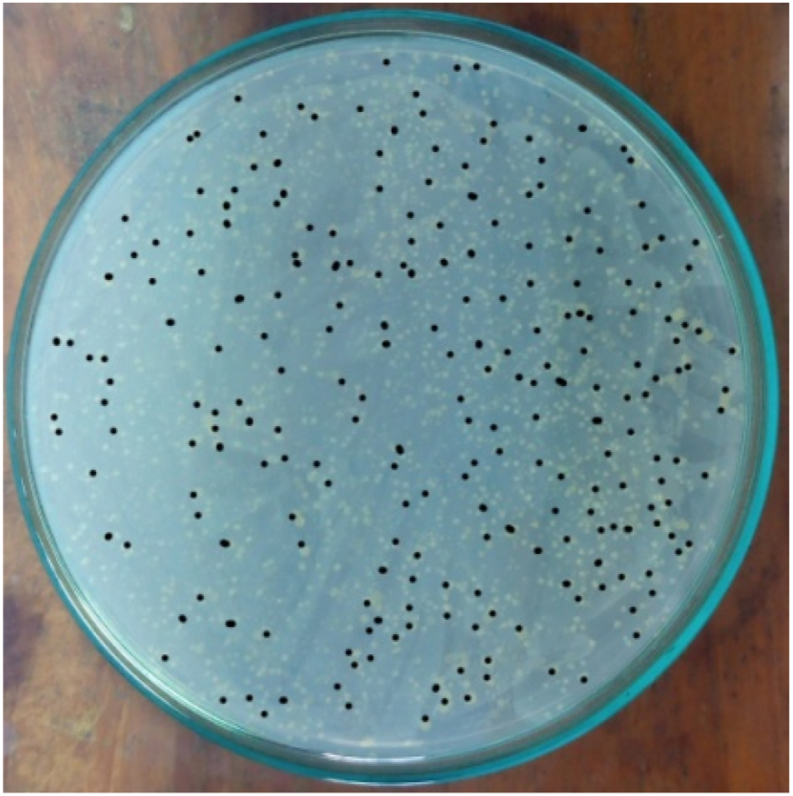
Bacterial growth inhibition by Miana or Quercetin and/or Cefixime intervention. Colony growth of S. Typhi after distilled water treatment as the negative control.

The bacterial load was quantified by counting CFUs on each plate. The bacterial load was quantified using the following formula: bacterial load (CFU/ml) = (number of CFUs on plate × 10^3^)/dilution [26]. A total of 10^3^ bacteria were injected intraperitoneally, and 10^7^ bacteria were found in the peritoneal fluid 24 h later. In the negative control group the number of bacteria could not be counted, on the other hand in the M or Q group combined with C no bacterial growth was found, similarly in the C only group no bacteria were found. Whereas the M, Q or M + Q combination group found bacterial growth in 3 of 5 plates, 3 of 5 plates and 2 of 5 plates, respectively at the end of the observation (Fig. 3, Fig. 4, Fig. 5, Fig. 6, Fig. 7). Bacterial growth inhibition by M or Q and/or C intervention was shown colony growth of S. Typhi after M (Fig. 3); colony growth of S. Typhi after Q (Fig. 4) and colony growth after M + Q intervention (Fig. 5); No growth of S. Typhi after Cefixime (C) or Miana + (C) or Q + C or M + Q + C intervention (Fig. 6); and colony growth of S. Typhi after distilled water treatment in the all plate of negative control group (Fig. 7).

## 4 Discussion

S. Typhi is a bacterium that causes infection in the digestive tract and can attack other organs due to systemic infection [21].

NF-κB can be activated in the presence of oxidation stress which will induce Reactive Oxygen Species (ROS) and affect the activation of NF-κB and HIF-1α in hypoxic conditions due to microorganism infection and doxorubicin induction in cardiac failure [12,28,29]. NF-κB plays a role in bacterial infection, where bacteria or bacterial components can activate NF-κB and signaling molecules at the initial of infection and then NF-κB will strengthen and ensure that the inflammatory process runs continuously resulting in pro-inflammatory cytokines and tissue hypoxia [30,31].

Miana (*Coleus scutellariodes*) is a famous herbal medicine in Indonesia used as an antimicrobial drug [[32], [33], [34]], antioxidant [18], antiangiogenesis [17] and anti-inflammatory [6,34] with the main phytochemical components being flavonoids, steroids, tannins, saponins, alkaloids [7,8,35].

Quercetin is a pigment of plant containing flavonoids which are strong antioxidant compounds and using nanoformulation or liposomal formulas allows it to be used in drugs in various diseases and may contribute to the prevention of atherosclerosis and cardiovascular disease [36].

Given the fact that Quercetin and Miana are widely employed in herbal medicine, further study is required to understand the molecular and immunology mechanisms underlying their anti-inflammatory, antibacterial, and dynamics of NF-κB alterations in S. Typhi infections. Study on Quercetin and Miana in relation to NF-κB activity is therefore required as a potential anti-inflammatory and antibacterial mechanism in S. Typhi infection.

The results show that decreased NF-κB mRNA expression, NF-κB protein levels, and bacterial load after administration of M + C (as an antibiotic agent), Q + C or the combination of M + Q + C showed significant differences when compared to sterile distilled water. The decline in NF-κB was stronger when the combination of M + Q + C was given compared to M, Q, M + Q or C only. The effects of NF-κB suppression appear to be the same between the administration of M, Q and the M + Q when C is added. However, the suppression of NF-κB. was not significant without adding C. Also, the suppression of NF-κB is in line with the bacterial load, so it can be concluded that both Quercetin and Miana only have bacteriostatic and not bactericidal effects. Here clearly shows that both Quercetin and Miana are used as a supplement in infections caused by S. Typhi. The results of this study indicate that Quercetin and Miana used as potential anti-inflammatory in S. Typhi infection by suppressing NF-κB activity. Miana shown to prevent the growth of *Candida albicans* [6], *M. tuberculosis* [17,18], *Klebsiella pneumoniae* [19], and S. Typhi in vitro [31]. All of these studies came to the same conclusions as our study, which concluded that Miana has bacteriostatic effects alone and no bactericidal ones. Quercetin and Miana can therefore be added to existing treatments for S. Typhi infection. Additionally, an in vivo study on Miana demonstrated an anti-inflammatory impact following infection of bacteria as well as a reduction in the levels of IL37, VEGF, HIF-1, and Intracellular Adhesion Molecule-1 (ICAM-1). This is consistent with the findings of the study, which showed that Miana suppresses NF-κB signaling during the inflammatory process [6,17,18].

This study's limitations still require further study into the intricate dynamics and relationship between NF-κB activity and other inflammatory and hypoxic processes like ROS, HIF-1, and VEGF, which will lead to dysfunction of several organs.

## 5 Conclusion

Apart from Cefixime, antibiotics used to treat S. Typhi infections, Quercetin, and Miana have the potential used as strong anti-inflammatory agents that inhibit NF-κB activity and contribute to the reduction of the inflammatory process.

## Ethic approval

The research was carried out after obtaining approval for ethical clearance from the health research ethics committee of the Faculty of Medicine UNHAS Makassar, Indonesia, No 306/UN4.6.4.5.3U PP36/2023, dated May 12, 2023 according to Helsinki Declaration.

## Funding

No specific funding are available.

## Consent for publication

Not applicable.

## Availability of data and materials

Data included in article/supp. material/referenced in article.

## Open Access

This is an Open Access article that uses a funding model which does not charge readers or their institutions for access and distributed under the terms of the Creative Commons Attribution License (http://creativecommons.org/licenses/by/4.0) and the Budapest Open Access Initiative (http://www.budapestopenaccessinitiative.org/read), which permit unrestricted use, distribution and reproduction in any medium, provided the original work is properly credited.

## CRediT authorship contribution statement

**Ade Rifka Junita:** Writing – review & editing, Writing – original draft, Validation, Software, Methodology, Investigation, Formal analysis, Data curation, Conceptualization. **Firdaus Hamid:** Writing – review & editing, Writing – original draft, Validation, Supervision, Software, Methodology, Investigation, Formal analysis, Data curation, Conceptualization. **Budu Budu:** Writing – review & editing, Writing – original draft, Validation, Project administration, Methodology, Investigation, Formal analysis, Data curation, Conceptualization. **Rosdiana Natzir:** Writing – review & editing, Writing – original draft, Validation, Supervision, Investigation, Formal analysis, Data curation, Conceptualization. **Yusmina Hala:** Writing – review & editing, Writing – original draft, Visualization, Validation, Software, Project administration, Investigation, Formal analysis, Data curation, Conceptualization. **Gemini Alam:** Writing – review & editing, Writing – original draft, Resources, Methodology, Investigation, Formal analysis, Data curation, Conceptualization. **Rosana Agus:** Writing – review & editing, Writing – original draft, Validation, Supervision, Software, Methodology, Formal analysis, Data curation, Conceptualization. **Burhanuddin Bahar:** Investigation, Formal analysis, Data curation, Conceptualization. **Ahmad Syukri:** Writing – review & editing, Writing – original draft, Software, Investigation, Formal analysis, Data curation, Conceptualization. **Muhammad Reza Primaguna:** Writing – review & editing, Writing – original draft, Validation, Software, Methodology, Investigation, Data curation. **Ressy Dwiyanti:** Writing – review & editing, Writing – original draft, Software, Methodology, Formal analysis, Data curation, Conceptualization. **Andini Febrianti:** Writing – review & editing, Writing – original draft, Software, Investigation, Formal analysis, Data curation, Conceptualization. **Muhammad Sabir:** Writing – review & editing, Validation, Software, Methodology, Formal analysis, Data curation, Conceptualization. **Azhar Azhar:** Writing – review & editing, Writing – original draft, Validation, Methodology, Formal analysis, Conceptualization. **Mochammad Hatta:** Writing – review & editing, Writing – original draft, Validation, Supervision, Resources, Methodology, Investigation, Formal analysis, Data curation, Conceptualization.

## Declaration of competing interest

The authors declare that they have no known competing financial interests or personal relationships that could have appeared to influence the work reported in this paper.
