# Supplementary material for: A potential mechanism of miana (Coleus scutellariodes) and quercetin via NF-κB in Salmonella typhi infection
Source: Heliyon. 2023 Nov 14;9(11):e22327. doi: 10.1016/j.heliyon.2023.e22327 (PMC10696054; doi:10.1016/j.heliyon.2023.e22327)
Supplement: Multimedia component 1 [file mmc1.pdf]

Supplement 1. List Sample and RT PCR Results

No	Group	1		2		3	
		RTPCR		RTPCR		RTPCR	
		No	Result (Fold Change)	No	Result (Fold Change)	No	Result (Fold Change)
1	I. Miana (M)	LA01	6.515	LB01	12.443	LC01	9.078
2		LA02	6.109	LB02	11.984	LC02	10.403
3		LA03	7.172	LB03	11.751	LC03	10.702
4		LA04	5.918	LB04	12.077	LC04	10.634
5		LA05	5.235	LB05	11.512	LC05	9.787
6	II. Quercetin (Q)	LA06	6.007	LB06	13.006	LC06	10.287
7		LA07	6.842	LB07	12.637	LC07	10.865
8		LA08	6.718	LB08	11.025	LC08	9.862
9		LA09	5.014	LB09	12.068	LC09	10.580
10		LA10	6.494	LB10	12.291	LC10	9.577
11	III. M + Q	LA11	6.133	LB11	11.887	LC11	10.071
12		LA12	5.880	LB12	11.226	LC12	9.975
13		LA13	6.957	LB13	13.152	LC13	10.772
14		LA14	5.514	LB14	11.959	LC14	9.614
15		LA15	7.116	LB15	12.979	LC15	10.329
16	IV. M + Cefixime (C)	LA16	5.281	LB16	12.724	LC16	8.367
17		LA17	5.479	LB17	12.583	LC17	7.645
18		LA18	6.278	LB18	12.434	LC18	8.064
19		LA19	5.699	LB19	11.498	LC19	7.905
20		LA20	6.501	LB20	12.891	LC20	7.825
21	V. Q + C	LA21	5.323	LB21	11.508	LC21	8.206
22		LA22	6.990	LB22	11.356	LC22	8.147

23		LA23	5.820	LB23	12.313	LC23	7.414
24		LA24	6.271	LB24	11.558	LC24	7.766
25		LA25	6.514	LB25	12.190	LC25	7.881
26	VI. M + Q + C	LA26	6.080	LB26	12.546	LC26	8.082
27		LA27	6.890	LB27	12.404	LC27	8.377
28		LA28	5.378	LB28	11.079	LC28	7.620
29		LA29	6.477	LB29	12.831	LC29	7.485
30		LA30	6.709	LB30	12.291	LC30	8.287
31	VII. Cefixime (Positive Control)	LA31	5.645	LB31	13.067	LC31	8.416
32		LA32	5.255	LB32	12.013	LC32	8.910
33		LA33	7.196	LB33	11.614	LC33	8.360
34		LA34	6.180	LB34	13.179	LC34	8.204
35		LA35	6.656	LB35	12.670	LC35	8.613
36	VIII. Destilled water (Negative Control)	LA36	7.064	LB36	11.231	LC36	15.159
37		LA37	6.351	LB37	12.717	LC37	14.222
38		LA38	5.885	LB38	11.890	LC38	14.922
39		LA39	6.944	LB39	11.457	LC39	14.470
40		LA40	7.145	LB40	11.965	LC40	14.373

Note Sample:
1. Before infection
2. After S. Typhi infection
3. 14 days after infection
