# Supplementary material for: A potential mechanism of miana (Coleus scutellariodes) and quercetin via NF-κB in Salmonella typhi infection
Source: Heliyon. 2023 Nov 14;9(11):e22327. doi: 10.1016/j.heliyon.2023.e22327 (PMC10696054; doi:10.1016/j.heliyon.2023.e22327)
Supplement: Multimedia component 3 [file mmc3.pdf]

Supplement 3. CFU of each animal according to experimental group

Group	Sample No	CFU
**Miana**	K01	0 (no colony)
	K02	0 (no colony)
	K03	17
	K04	19
	K05	18
**Quercetin**	K06	0 (no colony)
	K07	19
	K08	18
	K09	20
	K10	0 (no colony)
**Miana + Quercetin**	K11	16
	K12	0 (no colony)
	K13	0 (no colony)
	K14	17
	K15	0 (no colony)
**Miana + Cefixime**	K16	0 (no colony)
	K17	0 (no colony)
	K18	0 (no colony)
	K19	0 (no colony)
	K20	0 (no colony)
**Quercetin + Cefixime**	K21	0 (no colony)
	K22	0 (no colony)
	K23	0 (no colony)
	K24	0 (no colony)
	K25	0 (no colony)
**Miana + Quercetin + Cefixime**	K26	0 (no colony)
	K27	0 (no colony)
	K28	0 (no colony)
	K29	0 (no colony)
	K30	0 (no colony)
**Cefixime (Positive Control)**	K31	0 (no colony)
	K32	0 (no colony)
	K33	0 (no colony)
	K34	0 (no colony)
	K35	0 (no colony)
**Distilled water (Negative control)**	K36	ND (difficult to count)
	K37	ND (difficult to count)
	K38	ND (difficult to count)
	K39	ND (difficult to count)
	K40	ND (difficult to count)
